# Asthma and COVID-19: Emphasis on Adequate Asthma Control

**DOI:** 10.1155/2021/9621572

**Published:** 2021-08-24

**Authors:** David D. Nassoro, Leodegard Mujwahuzi, Issakwisa Habakkuk Mwakyula, Mwajabu K. Possi, Sylvester L. Lyantagaye

**Affiliations:** ^1^Department of Internal Medicine, University of Dar Es Salaam, Mbeya College of Health and Allied Sciences, Mbeya, Tanzania; ^2^Department of Internal Medicine, Mbeya Zonal Referral Hospital, Mbeya, Tanzania; ^3^Department of Social Sciences, University of Dar Es Salaam, Mbeya College of Health and Allied Sciences, Mbeya, Tanzania; ^4^Department of Biochemistry and Molecular Biology, University of Dar Es Salaam, Mbeya College of Health and Allied Sciences, Mbeya, Tanzania

## Abstract

Asthmatics are at an increased risk of developing exacerbations after being infected by respiratory viruses such as influenza virus, parainfluenza virus, and human and severe acute respiratory syndrome coronaviruses (SARS-CoV). Asthma, especially when poorly controlled, is an independent risk factor for developing pneumonia. A subset of asthmatics can have significant defects in their innate, humoral, and cell-mediated immunity arms, which may explain the increased susceptibility to infections. Adequate asthma control is associated with a significant decrease in episodes of exacerbation. Because of their wide availability and potency to promote adequate asthma control, glucocorticoids, especially inhaled ones, are the cornerstone of asthma management. The current COVID-19 pandemic affects millions of people worldwide and possesses mortality several times that of seasonal influenza; therefore, it is necessary to revisit this subject. The pathogenesis of SARS-CoV-2, the virus that causes COVID-19, can potentiate the development of acute asthmatic exacerbation with the potential to worsen the state of chronic airway inflammation. The relationship is evident from several studies that show asthmatics experiencing a more adverse clinical course of SARS-CoV-2 infection than nonasthmatics. Recent studies show that dexamethasone, a potent glucocorticoid, and other inhaled corticosteroids significantly reduce morbidity and mortality among hospitalized COVID-19 patients. Hence, while we are waiting for more studies with higher level of evidence that further narrate the association between COVID-19 and asthma, we advise clinicians to try to achieve adequate disease control in asthmatics as it may reduce incidences and severity of exacerbations especially from SARS-CoV-2 infection.

## 1. Introduction

Asthma is a significant, independent risk factor for developing lower respiratory tract infection [1–4], including viral pneumonia [5–8]. The observed increased incidence is generally due to relative immune dysfunction against various etiologies observed in asthmatics [9–11]. Asthma exacerbations are commonly seen in the pediatric population; however, they can also occur in adults and are precipitated mainly by viral infections [12]. Despite rhinovirus being the commonest infectious etiology for asthma exacerbation, virtually all other respiratory viruses can cause exacerbations [13]. In addition, bacterial etiologies can also significantly contribute to worsening asthma control, as shown in one meta-analysis study that concluded that asthma is a significant risk factor for developing invasive pneumococcal disease [14]. Thus, reducing exacerbation triggers is one of the most critical variables in achieving disease control, and currently, it is the main target in the management of asthma [15].

According to the recent Global Initiative for Asthma (GINA) update, asthma is a heterogeneous disease, usually characterized by chronic airway inflammation [16]. An asthma diagnosis is generally reached after a patient presents with respiratory symptoms such as wheeze, shortness of breath, chest tightness, and cough that vary over time and intensity, with variable expiratory airflow limitation that reverses upon using *β*_2_ adrenergic agonist [16]. According to the WHO, in 2019, the prevalence of asthmatics worldwide was 262 million, and, in that year alone, asthma contributed to 461,000 deaths [17].

Some countries have succeeded in reducing asthma exacerbation and hospitalization; however, in developing countries, the adverse clinical outcomes have increased by more than 30% in the past 20 years [18, 19]. The negative impact of asthma is widely felt by patients and relatives and by society and the healthcare system. Patients with persistent disease tend to experience more episodes of exacerbations, including recurrent pneumonia, compared with patients who are well controlled [20–23].

At the end of 2019, a novel coronavirus was implicated as an etiology of a cluster of pneumonia cases in Wuhan, Hubei Province in China. In February 2020, the World Health Organization (WHO) coined the disease caused by severe acute coronavirus 2 (SARS-CoV-2) coronavirus disease 2019 (COVID-19) [24]. It spread rapidly at an alarming rate throughout the world. In mid-march of 2020, the WHO recognized the illness as pandemic [25, 26]. The disease's symptomatic presentation varied widely; however, the primary clinical manifestation is mainly confined within the respiratory system with variable systemic responses depending on severity. The majority of symptomatic cases are due to pneumonia, while critical cases are mainly due to immune hyperactivation leading to cytokine storm [27–31].

Some of the risk factors for severe disease and death are older age, cardiovascular disease, diabetes mellitus, chronic respiratory diseases, hypertension, and cancer [32]. In one of the initial epidemiological studies to elucidate the risk factors for mortality in patients with COVID-19 in New York in 2020, asthma was not shown to be an independent risk factor for hospitalization; however, chronic obstructive pulmonary disease was significantly associated with mortality [33]. Furthermore, a recent GINA update on asthma and COVID-19 concluded that according to currently available evidence, asthmatics do not appear to be at an increased risk of acquiring COVID-19, and well-controlled and mild to moderate asthmatics did not appear to be at an increased risk of severe COVID-19; however, the risk for mortality was increased in asthmatics who recently needed inhaled corticosteroids and hospitalized patients with severe asthma [34, 35]. Additionally, in early 2020, The United States CDC advised asthmatic patients with moderate to severe illness to take extra measures to protect themselves as there is a risk of severe manifestation of COVID-19 [32].

A 2004 GINA survey found that the prevalence of asthmatics worldwide was 300 million, and it is expected to rise to more than 400 million by 2025 [36]. The prevalence of asthma varies from one region to another, and it is highly dependent on socioeconomic factors, wherein developing countries, health-seeking behavior and asthma case identification are poor [37]. Especially in developing countries, asthma is underdiagnosed and inadequately managed, leading to a higher prevalence of asthmatics who are poorly controlled [38, 39]. In developed countries, the prevalence of asthma ranges from 7% of the total population in France to around 15%–18% in the United States [40]. A systematic analysis that estimated the prevalence of asthma in Africa found that by 2010, 12.8% of Africans had asthma [38]. However, for the reasons discussed above, the prevalence in Africa may be significantly higher because of various reasons leading to underdiagnosis. A 2006 to 2008 behavioral risk factor surveillance system conducted by the CDC found that almost 64% of asthmatics in the United States had a persistent disease [41]. Furthermore, evidence during the early days of COVID-19 showed that asthmatics are overrepresented among hospitalized COVID-19 patients [35, 42, 43]. Asthma exacerbations are primarily due to poor disease control; therefore, due to the overwhelming burden of poorly controlled asthmatics globally, it is essential to revisit this topic, especially when we are facedwith a virulent respiratory virus pandemic[44–47].

## 2. Asthma Disease Control and Risk of Exacerbation

The accepted definition of asthma control and severity is arbitrary. However, a joint task force of the American Thoracic Society (ATS) and European Respiratory Society (ERS) manage to propose definitions of the terms. According to the task force, asthma severity is defined as the difficulty in controlling asthma with treatment after excluding modifiable factors such as poor adherence, smoking, and comorbidities. Severity primarily reflects the required level of treatment and the underlying disease state's activity during treatment. At the same time, asthma control encompasses not only the patient's current clinical state (symptoms, night waking, reliever use, and lung function) but also considers their “future risk”—that is, their potential for experiencing adverse outcomes, such as loss of control in the near or distant future, exacerbations, accelerated decline in lung function, or treatment-related side effects. It is emphasized that even if current poor control predicts future poor control and healthcare utilization, other pathologic and physiologic measures, independent of the level of current clinical control, can influence future risk [48].

The above definitions are relatively more practical in day-to-day practice; however, GINA came with a rather exhaustive list of variables to approach the meaning of control [49]. Thus, asthma is considered controlled if all of these features were present: diurnal symptoms less than once a week and no asthma attacks in the last three months, no activity (work or other activities) limitations in the last 12 months, no nocturnal symptoms in the last three months, short-acting *β*_2_ agonist twice or less per week in the last three months and no use of oral steroids in the last 12 months, and forced expiratory volume in one second (FEV_1_) of 80% of predicted value or greater. Moreover, asthma is considered partly controlled if one or two of the above features were absent and uncontrolled if more than two features were absent or if asthma, shortness of breath, or wheezing had caused hospital/emergency department admissions in the last 12 months; oral steroids were used in short courses of continuously in the last 12 months; or the subject had more than 12 attacks (1 per week or more) in the last three months.

In a large surveillance study done in the United States from 2006 to 2010, the Behavioral Risk Factor Surveillance System (BRFSS) that surveilled almost 10,000 and 52,000 children and adults with asthma, respectively, found that more than 38% of asthmatic children and 50% of asthmatic adults were uncontrolled [50]. Furthermore, a large surveillance study done in 10 countries in Europe found that 49% of adult asthmatics were poorly controlled [49]. In Asia, a large cross-sectional survey study found that more than 50% of patients experienced daytime symptoms; however, the information on the extent of disease control in the study was lacking [39]. Data estimating the extent of disease control in other regions containing developing countries is scarce; however, we can extrapolate asthma control in patients who reside in a region like Africa; specifically, sub-Saharan Africa will be poor, probably worse than in developed countries; this may be because of various challenges such as underdiagnosis of childhood asthma, inadequate asthma management, poor access to care, poor availability and relative prohibitive cost of inhaled medications, impaired control of environmental and potential triggers, inadequate disease education from healthcare providers, cultural and language barriers [51].

Asthma control is preferred instead of grading severity because several cross-sectional surveys from various countries have shown that the use of either inhaled corticosteroids (ICS) or rapid-acting reliever medications is independent of asthma severity [39, 52]. For example, a European survey of patients with persistent asthma symptoms showed that ICS were used by only 25% of adults with severe asthma, 23% with moderate asthma, and 28% with mild asthma. For children, the corresponding figures were 26%, 33%, and 33%, respectively [52]. Similar findings were recorded in a study of 3207 patients in Asia who participated in the Asthma Insight and Reality in Asia-Pacific (AIRAP) study [39].

Asthma exacerbation is one of the predictors of poorly controlled asthma; however, it cannot define it. Several factors can predict asthma exacerbations; some are pediatric patients, lower socioeconomic status, poor day-to-day asthma control, and active airway inflammation, especially if it is refractory to inhaled steroids [53]. However, respiratory infections, especially viral, are implicated in more than 80% of exacerbations, with rhinovirus (RV) infections responsible for more than 66% of cases [54, 55]. Lower respiratory tract symptoms, decreased lung function, and bronchial hyper-responsiveness are more prevalent in asthmatics with rhinovirus infection than in controls, and viral load correlates positively with the severity of symptoms [56]. Pathogenesis of rhinovirus is similar to SARS-CoV-2; however, the extent of inflammation due to COVID-19 is significantly more profound. Similar to COVID-19, RV induces inflammation where inflammatory cells such as neutrophil, eosinophil, CD4+ and CD8+, lymphocytes and mast cells are either activated or attracted to the area of infection in the airway parenchyma. Furthermore, there is an increase in the production of cytokines such as IL-6, IL-8, IL-16, INF-*α*, and interferon-gamma-induced protein 10 (IP-10) [57]. These proinflammatory cytokines can lead to airway hyper-responsiveness, inflammation, and increased mucus secretion [58].

## 3. Glucocorticoids as an Essential Drug for Asthma Disease Control

Several drugs are used for asthma control; however, in recent times, ICS have been the drugs of choice principally by reducing inflammation and increasing tolerance to triggers, especially in allergic asthma than nonallergic asthma [59, 60]. Additionally, several studies have shown that they are critical in achieving disease control in asthmatics [61–63]. ICS, even though not all patients may have access to obtain them, especially in developing countries, are widely available in many parts of the world [64]. The first ICS was beclomethasone dipropionate introduced in the early 1970s and was a revolutionary therapy in controlling asthma exacerbation while causing few adverse events compared with systemic glucocorticoids commonly used at the time [65]. By far, ICS are the most effective therapy used as controllers in asthmatics, and their use is recommended by reputable guidelines worldwide [61, 66, 67]. Some of the commonly used ICS are budesonide, beclomethasone, and fluticasone [68].

Pharmacological effects of glucocorticoids result from gene expression from binding the glucocorticoid receptor (GR), a member of the superfamily of nuclear receptors in the cytoplasm. Therefore, the overall outcome is either the promotion or inhibition of transcription of the respective genes [69]. Different mechanisms that were previously used to assess the potency of glucocorticoids did not account for the target organ difference in potency, and the suppressive effect on the adrenal gland may not necessarily reflect the anti-inflammatory action [70]. Therefore, it was essential to rank the ICS' potency based on their anti-inflammatory actions in the lungs; one study that assessed transactivation and transrepression of respiratory mediators came with the rank order that shows fluticasone propionate > budesonide > beclomethasone dipropionate, triamcinolone acetonide, and flunisolide [71]. ICS have broad anti-inflammatory effects in asthmatics and are effective in many patients with persistent asthma [72, 73]. Hence, ICS function mainly by reducing asthma symptoms, improving peak expiratory flow rate and quality of life. Furthermore, they diminish airway hyper-responsiveness and prevent or reduce the frequency of exacerbations [74].

Several studies have shown that ICS in asthmatics and COPD patients can increase the risk of upper respiratory tract infection and pneumonia [75–77]. *In vitro* studies have proposed that ICS may impair innate immune responses and reduce viral clearance [78–80]. The above observationsare not universal as other studies did not find an association between ICS and high incidence of pneumonia in asthmatics [81, 82]. Therefore, they underpin the fact that the use of corticosteroids, especially in viral infections, is still debatable and should be individualized based on the viral etiology in question and the stage or severity of the disease.

## 4. Glucocorticoids and COVID-19

Even before the emergence of SARS-CoV-2 in late 2019, there exists evidence to suggest the added benefit of using corticosteroids in viral infections, specifically those due to coronaviruses. An early study found that pretreatment of human respiratory cells *in vitro* with budesonide, combined with glycopyrronium and formoterol, inhibited human coronavirus's (HCoV-229E) replication capacity and was associated with the reduction of cytokine production [83]. Recently, preliminary studies that used intravenous hydrocortisone or dexamethasone in patients with moderate to severe COVID-19 were halted early after the data suggested statistical, clinical benefit [84, 85]. In a large clinical trial, the RECOVERY trial, published in early 2021, 2104 and 4321 hospitalized patients with COVID-19 were assigned to receive either oral or intravenous dexamethasone 6 mg once daily for up to 10 days or usual care. 482 patients (22.9%) in the dexamethasone group and 1110 patients (25.7%) in the usual care group died within 28 days after randomization (age-AOR = 0.83; 95% CI [0.75–0.93]; *P* < 0.001) [86]. With these findings, the study recommended dexamethasone for treating patients with COVID-19 who are on supplemental oxygen, and if unavailable, other corticosteroid equivalents, such as hydrocortisone, methylprednisolone, and prednisolone, may be used [87].

A significant randomized controlled trial published in early 2021 that assessed the clinical outcomes of hospitalized patients with COVID-19 in the aspect of the use of ICS in asthmatics and COPD patients in the United Kingdom showed that in patients 50 years and older, ICS use in asthmatics was associated with lower mortality than in those patients without an underlying respiratory condition [34]. Furthermore, in a similar randomized controlled trial where all adults irrespective of asthma diagnosis were initiated on either inhaled budesonide or placebo within seven days from the onset of mild COVID-19 symptoms in addition to conventional treatment, primary outcomes of COVID-19-related urgent care visit or hospitalization were reduced to 3% in the ICS group as opposed to 15% in the conventional treatment group [88]. Therefore, these findings suggest that besides playing a significant part in achieving adequate asthma control, ICS use in asthmatics may be crucial in reducing adverse clinical outcomes following COVID-19 diagnosis. Some of the important studies that analyzed the importance of steroids in SARS-CoV-2 infection are summarized in Table 1.

## 5. The State of Relative Immune Dysfunction in Asthmatics

Most of the research done in asthmatics is directed toward explaining the causes, prognosis, and therapy, while few studies have been done that show the pathogenesis of asthma as one of the significant risk factors of pneumonia. One of the mechanisms of immune dysfunction may be the increased risk of infection due to opportunistic pathogens secondary to airway inflammation in patients whose disease is poorly controlled. In a proportion of asthmatics, dysfunction of innate, humoral, and cell-mediated immunity (CMI) has been an essential contributing factor for the observed increased incidence of microbial infections, especially in the respiratory system.

Innate immune dysfunction and impaired T-helper type 2 (Th2) response at the level of epithelial cells can occur in patients with asthma [56, 89, 90]. The discovered mechanism is pertinent to the increased risk of viral infections by the impaired secretion of interferon beta (INF-*β*) and INF-*λ* by bronchial epithelial cells and bacterial infection by the impairment of toll-like receptor 2-mediated signal transduction recruiting neutrophils in atopic patients [9, 91, 92]. Additionally, several studies have found evidence of impairment of humoral immune response in patients with atopic conditions such as asthma that contribute to an increase in susceptibility to microbial infection and their overall progression and adverse clinical outcomes. For example, an early randomized control study that assessed the response of asthmatic and control subjects to tetanus toxoid found that 13 of 74 asthmatic patients (18%) and 1 of 74 controls (1.3%) had no response (*P* < 0.001) [93]. Another study conducted in patients with atopic dermatitis yielded similar results [94]. Moreover, a study comparing antibody levels of pneumococcal vaccine (PPV-23) in children aged 2 to 18 years found that patients with asthma had significantly lower antibody levels than children without asthma both before and after vaccination [95].

Additionally, cell-mediated immunity (CMI) in a subset of asthmatics can be impaired. The exact mechanism of impairment of CMI in this patient population is still poorly understood. One of the early studies to show the dysfunction of CMI in asthmatics was done by Grove et al. and reported that 9.2% and 1.2% of asthmatics and controls, respectively, had no delayed-type hypersensitivity response to any of fungal, viral, and tuberculous antigens (*P* < 0.02) [93]. Similar findings were found in patients with atopic [94]. In Japan, a study found an inverse relationship between the delayed hypersensitivity reaction to *Mycobacterium tuberculosis* and atopy [96]. Furthermore, patients with atopic dermatitis complicated with herpes simplex virus (HSV) infection had significantly fewer INF-*γ* spot forming cells and less secretion of INF-*γ* by peripheral blood mononuclear cells stimulation by HSV [97]; this may suggest that impairment of CMI alone may contribute to the development of severe eczema herpeticum. Similar to humoral immunity impairment in asthmatics, the development of CMI may be impaired by the decreased response to epitopes or pathogen-specific rapid waning of the immunity.

## 6. Possible Pathogenic Relationship between Asthma and COVID-19

Mast cells are at the center of asthma pathogenesis especially Th2 type; similarly, they also play a significant role in the pathogenesis of SARS-CoV-2, especially in inducing and sustaining inflammation [30, 98–100]. In addition, other inflammatory cells such as macrophages, basophil, and T lymphocytes play essential roles in both diseases; however, their respective contribution in poorly controlled asthmatics infected with SARS-CoV-2 may be limited. Therefore, it is logical to overview individual pathogenesis and analyze the possible ways the SARS-CoV-2 infection contributes to asthma exacerbation.

### 6.1. Overview of Asthma Pathogenesis

Asthma's pathogenesis is mediated by deranged response to common allergens by eliciting an exuberant inflammatory response and its subsequent effect in lung parenchymal tissues, specifically the airway smooth muscles. Therefore, airway hyper-responsiveness is accompanied by enhanced sensory irritability of the airways and increased mucus secretion.

Dendritic cells process the inhaled allergen and migrate to local lymphoid collections, and through MHC class II, antigen presentation occurs. Depending on dendritic cells' potency to synthesize IL-12, the balance between Th1 and Th2 responses is realized by favoring the Th1 response [101]. Several studies have shown that Th1 responses become more apparent with asthma severity [102–104]. Thus, the Th1/Th2 imbalance may explain the tissue-damaging aspects of severe asthma. In addition, Th1 cells and CD8+ cells have also been implicated in asthma exacerbation, especially following viral infection; however, the exact mechanism is still debatable [105, 106]. Therefore, asthma's pathogenesis is generally driven by variable Th1/Th2 cellular responses [107].

Mast cells have long been associated with asthma, with early asthmatic reaction following allergen inhalation being mast cell-dependent. Drugs such as sodium cromoglycate and nedocromil sodium inhibit mast cell degranulation, further strengthening the association with asthma [108]. After sensitization by an allergen, Th2 promotes IgE production specific to that allergen and is eventually taken up by mast cells, which becomes the basis of initial allergic reaction in asthmatics [109]. Mast cells in the airway epithelium and submucosa are important in immediate allergic reactions such as bronchoconstriction and increased mucus secretion. In contrast, mast cells located deeper in the airway wall play an essential role in chronic inflammatory responses in asthmatics [98, 110, 111]. In chronic asthmatics, mast cell concentration is significantly increased in association with airway smooth muscle (ASM) in large and small airways [111]. At this site, mast cells interact with ASM through autacoid mediators that can induce fibrogenesis, hyperplasia, and hypertrophy of smooth muscles as part of remodeling response. This phase of cytokine secretion constitutes the late-phase postallergen sensitization. It comprises the recruitment of inflammatory cells that mainly participate in chronic inflammation such as eosinophils, basophils, neutrophils, and *T* helper and memory *T* cells to the allergen site. Monocytes and dendritic cells can also be recruited [112, 113].

### 6.2. Overview of COVID-19 Pathogenesis

Type II pneumocytes are the primary type of cells implicated in the pathogenesis of COVID-19. Furthermore, other cells with angiotensin convert enzyme-2 (ACE2) receptors, such as those located in the vascular epithelial and endothelial cells, cardiomyocytes, intestinal epithelia cells, renal epithelial cells, and proximal renal cells, can also be infected [114]. ACE2 mRNA expression is also high in the cerebral cortex, hypothalamus, striatum, and brainstem. Hence, this can explain symptoms such as anosmia and dysgeusia [115, 116]. The host's immune system is activated as a result ofpathogen antigens such as pathogen-associated molecular patterns (PAMPs) and as an injury or damage-associated molecular patterns (DAMPs) following SARS-CoV-2 infection of the susceptible cells [117–120]. DAMPs and PAMPs are recognized by neighboring cells such as epithelial cells, endothelial cells, and macrophages, leading to proinflammatory cytokines such as IL-6, interferon-gamma-induced protein 10 (IP-10), and macrophage inflammatory protein. These mediators attract monocytes, neutrophils, macrophages, and T cells at the infection site aggravating the inflammation [121]. These mediators are indicators of Th1 response similar to SARS-CoV-1 and MERS-CoV [122].

However, mast cells are also a key component of proinflammatory cytokines in COVID-19 because of infection from SARS-CoV-2 [112, 123–125]. Various viral components can activate mast cells through toll-like receptor type 3, which can recognize PAMP with an outcome of inducing cytokine production. Additionally, mast cells possess a significant amount of ACE2 receptors required for the binding of SARS-CoV-2 [99, 126]. Furthermore, following stimulation, mast cells rapidly secrete preformed granules-stored inflammatory mediators such as histamine, TNF-*α*, prostaglandin D_2_ (PGD_2_), and several other chemokines [127]. Acute inflammatory mediators are released immediately, while delayed inflammatory mediators are released progressively within 6 to 24 hours. Besides the contribution of type II pneumocytes in developing cytokine-storm, several studies have emphasized the importance of mast cells as an essential contributor to the pathology [30, 31, 99, 100].

### 6.3. The Implication of COVID-19 Pathogenesis in Asthmatics

One of the common routes of inflammation in COVID-19 and asthma, mainly if asthma is poorly controlled, is the activation of Th1 cells. In asthmatics, Th1 has a significant contribution in maintaining inflammation in the epithelial and subepithelial layers of the airway leading to swelling of the bronchial wall, increased nonspecific airway hyper-responsiveness, chronic state of inflammation, and sustained airway remodeling [98, 128, 129]. The activation of mast cells by SARS-CoV-2 infection can worsen asthma control in several ways. Mast cell-derived mediators, mainly histamine, leukotrienes, PGD_2_, PGE_2_, tumor necrosis factor (TNF), thymic stromal lymphopoietin (TSL), and tryptase, induce the classic features of early asthmatic reaction *in vivo,* therefore inducing bronchoconstriction, mucosal edema, and mucus hypersecretion and hence the observed worsening of asthma control in a subset of asthmatics infected with SARS-CoV-2 [128, 130, 131].

## 7. COVID-19 Incidences in Asthmatics

There is a significant regional variation on the prevalence of symptomatic COVID-19 asthmatic patients. In one of the earlier studies that assessed 140 hospitalized COVID-19 patients in Wuhan, China, no asthma cases were observed [132]. Another multicenter study included 476 patients and studied those patients' clinical characteristics; none of the patients were asthmatic [133]. In another systematic review of the prevalence of comorbidities in patients with COVID-19 in China, the prevalence of asthma among admitted patients was 1.5% [134]. Similar results were observed in Italy, whereby the prevalence of asthma among 1591 patients was very low such that it was difficult to assess if it was a significant risk factor.

A large national cross-sectional study conducted in China in 2019 concluded that asthma is significantly underdiagnosed and undertreated [135], with only 28% of asthmatics reporting to be diagnosed by a physician. Therefore, this may create a considerable bias in identifying risk factors for COVID-19, as the clinical presentation of COVID-19 may mask the clinical features of asthma in a previously undiagnosed patient. Furthermore, studies for Australia, Italy, and the United States have reported relatively different clinical features and functional characteristics [136–138]. Furthermore, it is common to find a varied degree of irreversible airway obstruction in the elderly population, complicating the diagnosis [139].

In contrast to China and Italy, asthma was a significant risk factor for morbidity and mortality of COVID-19 in other studies done in the United States and the United Kingdom. More than 9% of 5,700 hospitalized patients were asthmatic in one observational study in New York [140]. The prevalence in 16,749 admitted COVID-19 patients in the UK is even higher at 14%. Studies that assessed the asthmatic proportion that know their diagnosis in these countries are few. These critical observations may show that most asthmatics in these countries know their diagnosis and strengthen the known fact that asthma is a risk factor of pneumonia.

In another large-scale nationwide study with a cohort of 7340 COVID-19 patients, severe outcomes were observed in 6.9% and 3.7% of patients with and without asthma, respectively (adjusted OR = 1.62), and 4.7 and 3.7 of patients with and without allergic rhinitis, respectively (adjusted OR = 1.27; *P* < 0.05). Furthermore, individuals with nonallergic asthma had a higher risk of severe outcomes from COVID-19 than those with allergic asthma in the study [141, 142].

The inconsistent findings between asthma and the severity of COVID-19 may be because of patients' age, disease severity disparity, disease control disparity, racial disparities, differing criteria for hospitalization, and scale of study. In addition, few studies have reported adequate clinical information in asthmatics with COVID-19, such as lung function, control status, asthma phenotypes, and treatment regimen; therefore, in this scenario, comparing different studies becomes difficult.

## 8. Clinical Presentation of COVID-19 in Asthmatics

Although both asthma and COVID-19 are primarily diseases of the respiratory system, their respective clinical features can vary significantly; however, logically, in an asthmatic patient with exacerbation presenting with COVID-19, clinically, we expect to get a varied combination of clinical features contributed by the two diseases.

Clinical presentation of acute asthma exacerbation varies considerably, from mild form to dramatic presentation of acute severe asthma exacerbation (status asthmaticus), and the severity of the episode may change abruptly even during treatment [143]. Nevertheless, the symptomatology is quite similar with respect to the severity of the disease with episodes and extent of exacerbation presenting with various degrees of exaggeration of symptoms. The four most common symptoms in asthmatics are wheeze, cough, shortness of breath or difficulty breathing, and varied sensation of chest tightness [144]. On physical examination, high-pitched, musical wheezes are vital signs though not specific in asthmatics. Lower-pitched rhonchi could be heard in some patients during auscultation if wheezes were absent [143]. Additionally, crackles may also be heard during physical examination [145].

COVID-19 has a wide range of symptoms; however, the commonest symptoms are cough, usually dry, headache, fever, and myalgias. In addition, dyspnea, sore throat, diarrhea, nausea and vomiting, and loss of smell or taste are also frequently encountered in these patients [146]. A study that analyzed the characteristics of pulmonary auscultation in patients with laboratory-confirmed COVID-19 found that 73.7% of patients cough during auscultation; furthermore, using high-quality auscultation recordings, wheezes, and coarse crackles were the commonest findings [147]. Additionally, most cases had normal breath sounds in the upper lungs. However, the incidences of abnormal breath sounds were more prevalent toward the lung bases, especially the posterior of the chest.

The pathophysiology of respiratory failure in asthma and COVID-19 are quite different. In asthma, the main aspects are respiratory flow limitation, which is generally due to inflammation and bronchospasm; however, in COVID-19, vascular microthrombi, shunts, and alveolar exudate predominate [148–152]. Moreover, during mechanical ventilation, the pathophysiology of the underline disease process is thought to be heterogeneous. However, expiratory flow limitation is not commonly observed unless the patient has a concurrent obstructive pulmonary disease [153].

These clinical characteristics do not exclude the possibility of having COVID-19-related bilateral pneumonia in addition to an asthmatic exacerbation; therefore, we advise that both conditions, asthma and COVID-19, should be managed accordingly.

## 9. Conclusion and Recommendations

It is estimated that more than 60% of all asthmatics are poorly controlled and, therefore, at increased risk of acquiring lower respiratory infections that may further worsen the disease control. At the time when the world is faced with a respiratory virus with mortality several times as that of seasonal flu, we advise clinicians to try to achieve adequate disease control in asthmatic patients who are inadequately controlled. Moreover, asthmatics with COVID-19 may present with asthma exacerbations; hence, it is essential to perform a thorough clinical assessment in these patients and manage them accordingly. These approaches may prove vital in reducing morbidity and mortality in this population. However, further studies with a higher level of evidence are needed to elucidate the association between COVID-19 and asthma control, especially their respective contribution to morbidity and mortality.

## Figures and Tables

**Table 1:** Studies that evaluated the benefits of corticosteroids in COVID-19 patients.

Study, year	Aspect studied	Major findings
Yamaya et al., 2020 [83]	The inhibitory effects of LAMA glycopyrronium, LABA formoterol, and IC budesonide on HCoV-229E replication	Glycopyrronium, formoterol, and a combination of glycopyrronium, formoterol, and budesonide inhibit HCoV-229E replication partly by inhibiting receptor expression and/or endosomal function and that these drugs modulate infection-induced inflammation in the airway
Tomazini et al., 2020 [84]	To determine whether intravenous dexamethasone increases the number of ventilator-free days among patients with COVID-19-associated ARDS	Intravenous dexamethasone plus standard care, compared with standard of care alone, resulted in a statistically significant increase in the number of days alive and free of mechanical ventilation over 28 days (6.6 days vs 4.0 days)
The Writing Committee for the REMAP-CAP Investigators, 2020 [85]	To determine whether hydrocortisone improves outcome for patients with severe COVID-19	In this Bayesian randomized clinical trial that included 403 patients and was stopped early after results from another trial were released, treatment with a 7-day fixed-dose course of hydrocortisone or shock-dependent dosing of hydrocortisone, compared with no hydrocortisone, resulted in 93% and 80% probabilities of superiority, respectively, with regard to the odds of improvement in organ support-free days within 21 days
The RECOVERY Collaborative Group, 2020 [86]	To evaluate the effects of oral or intravenous dexamethasone in hospitalized with COVID-19	22.9% and 25.7% of patients in the dexamethasone group and usual treatment group, respectively, died within 28 days (adjusted odd rate ratio, 0.83; 95% CI [0.75–0.93], *P* < 0.001)
		Therefore, the use of dexamethasone resulted in lower 28-day mortality among those who were receiving either invasive mechanical ventilation or oxygen alone at randomization but not among those receiving no respiratory support
Ramakrishnan et al., 2021 [88]	To assess if inhaled glucocorticoids would be an effective treatment for COVID-19 patients within 7 days of onset of mild symptoms	14% and 1% in the usual group and budesonide group, respectively, experienced primary outcomes (difference on proportion, 0.131, 95% CI [0.043–0.218], *P*=0.004)
		Therefore, early administration of inhaled budesonide reduced the likelihood of needing urgent medical care and reduced time to recovery after early COVID-19.
